# 93. Biomarkers of Amphotericin-Induced Complement Activation and Associated Clinical Manifestations

**DOI:** 10.1093/ofid/ofae631.030

**Published:** 2025-01-29

**Authors:** George R Thompson, Gelina M Sani, Monica Donnelley, Jaimie Figueroa, Andre Milner, Juan Arredondo, Marie Nearing, Ikaika Christian Loque, Satya Dandekar

**Affiliations:** University of California Davis Medical Center, Sacramento, CA; UC Davis Health, Sacramento, CA; University of California Davis Medical Center, Sacramento, CA; UC Davis Health, Sacramento, CA; UC Davis Health, Sacramento, CA; UC Davis, Davis, California; UC Davis, Davis, California; UC Davis, Davis, California; UC Davis Health, Sacramento, CA

## Abstract

**Background:**

Therapeutic liposomes and lipid excipient medications are frequent causes of infusion reactions. Liposomal amphotericin B (L-AmB) is well-known to induce hypersensitivity reactions upon initial infusion. Animal models have suggested this phenomenon as secondary to a primitive immune response mediated by complements (complement activation-related pseudoallergy (CARPA). We performed a prospective study investigating the biologic response to L-AmB infusion to explore the pathophysiologic mechanism(s) responsible for infusion related adverse events.

**Figure 1.**
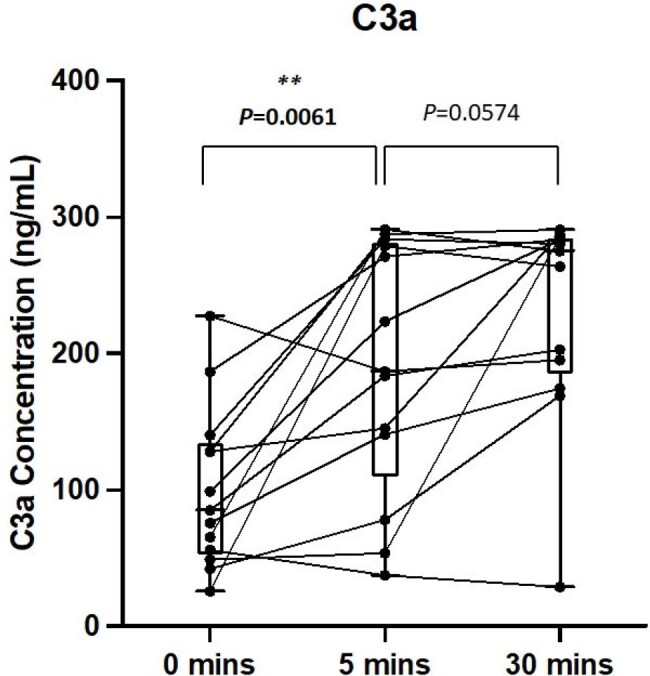
Day 0 C3a Concentrations

C3a concentrations were elevated 5 minutes after amphotericin infusion. C3a concentrations were measured by ELISA. Statistics performed by Wilcoxon matched-pairs signed rank test, comparing samples to baseline pre-infusion measurements, p = 0.0061.

**Methods:**

This is a single-center, prospective, observational, blood draw only study that includes patients 12 years of age and older who were admitted to a large academic medical center receiving initial L-AmB infusions. Excluded were those less than 12 years of age, those who had previous treatment history with L-AmB formulations, pregnant and incarcerated patients. Lab draws occurred prior to amphotericin infusion (time 0), 5 minutes after the start of infusion, and 30 minutes post-infusion. The samples were batched, transported to the lab for analysis (ELISA), and data output analyzed. The primary outcome of this study is the change in complement levels during and after amphotericin infusion.

**Figure 2.**
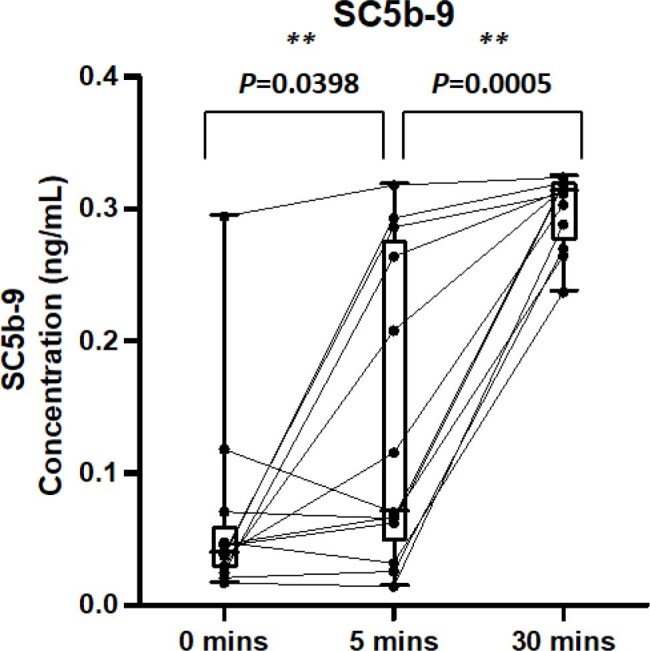
Day 0 SC5b-9 Concentrations SC5b-9 concentrations were elevated 5 minutes after amphotericin infusion and 30 minutes post infusion. SC5b-9 concentrations were measured by ELISA. Statistics performed by Wilcoxon matched-pairs signed rank test, comparing samples to baseline measurements. Pre-infusion to 5 minutes into the infusion p = 0.0061. 5 minutes into the infusion to 30 minutes post infusion p = 0.0005.

**Results:**

Thirteen patients were enrolled: mean age was 54.62 (± 16.99) and 10/13 (76.92%) had a comorbidity of malignancy. Following L-AmB infusion there was a clear increase in C3a at 5 min (P = 0.0061) and 30 minutes post infusion compared to time 0. Additionally, evaluation of SC5b-9 concentrations were also significantly higher 5 min into infusion (P = 0.0398) and 30 minutes post infusion (P = 0.0005). Among enrolled patients 5/13 received pre-medication with diphenhydramine and acetaminophen 30 minutes before their infusion and correlated with complement response.

**Conclusion:**

Clinical data confirms the likely mechanism of CARPA as responsible for L-AmB infusion reactions given the clear increase in C3a and SC5b-9 from baseline following infusion. These increases confirm prior findings in animal models found responsible for lipid-associated infusion reactions, and will assist in the development of future antifungal agents.

**Disclosures:**

**George R. Thompson, III, MD**, Astellas: Advisor/Consultant|Cidara: Advisor/Consultant|Cidara: Grant/Research Support|F2G: Advisor/Consultant|F2G: Grant/Research Support|Melinta: Advisor/Consultant|Melinta: Grant/Research Support|Mundipharma: Advisor/Consultant|Mundipharma: Grant/Research Support|Pfizer: Advisor/Consultant

